# Correction: Si et al. Dual-Targeted Extracellular Vesicles to Facilitate Combined Therapies for Neuroendocrine Cancer Treatment. *Pharmaceutics* 2020, *12*, 1079

**DOI:** 10.3390/pharmaceutics17101317

**Published:** 2025-10-11

**Authors:** Yingnan Si, JiaShiung Guan, Yuanxin Xu, Kai Chen, Seulhee Kim, Lufang Zhou, Renata Jaskula-Sztul, X. Margaret Liu

**Affiliations:** 1 Department of Biomedical Engineering, University of Alabama at Birmingham (UAB), 1825 University Blvd, Birmingham, AL 35294, USA; yingnan@uab.edu (Y.S.); guan0926@uab.edu (J.G.); yuanxin8@uab.edu (Y.X.); kaisdzb@uab.edu (K.C.); seulheekim@uabmc.edu (S.K.); 2 Department of Medicine, University of Alabama at Birmingham, 703 19th Street South, Birmingham, AL 35294, USA; lfzhou@uab.edu; 3 Department of Surgery, University of Alabama at Birmingham, 1808 7th Avenue South, Birmingham, AL 35294, USA; rjsztul@uabmc.edu

## Error in Figure

In the original publication [1], there was a mistake in Figure 3C as published. The Western blot image of QGP cell marker P21 was used on BON cells by mistake, which led to a duplicated result for the P21 marker. The corrected Figure 3C appears below.

## Text Correction

There was an error in the original publication [1]. The statement describing Figure 3C needs to be corrected accordingly since Figure 3C has been updated.

A correction has been made to Section 3.3. In Vitro Anticancer Cytotoxicity and Synergistic Mechanisms, Paragraph 2:

The Western blotting analysis of proliferation and apoptosis proteins showed that 10 nM Ver-A treatment significantly reduced the expression of proliferation signaling protein AKT (protein kinase B) and completely eradicated the expression of protein cyclin D1, P21 and P27 on BON cells (shown as sample 3). 5 nM Ver-A treatment did not impact the expression of AKT but did reduce cyclin D1 and P21 expression and almost eradicated P27 expression (shown as sample 2). Different from BON cells, the 5 or 10 nM Ver-A did not change the expression of AKT while completely blocked the expression of cyclin D1, p21 and p27 in QGP cells. These results are consistent with literature reporting that Ver-A downregulated the expression of Akt signaling proteins in pancreatic adenocarcinoma and prostate cancer [55,56]. Unlike Ver-A, it is found that 10 or 20 nM DM1 did not change the expression of AKT, cyclin D1, p21 and p27 in BON and QGP cells. In addition to free drugs, we also tested the EV-delivered combined 5 nM Ver-A and 5 nM DM1. Surprisingly, Bon and QGP cells treated with 5 nM EV-Ver-A/DM1 (sample 6) exhibited the same Western blot pattern as that treated with 10 nM free Ver-A drug. This is probably attributed to the better biocompatibility of EV, which leads to more efficient drug delivery than free drugs. In addition, unlike EV-encapsulated drugs, the hydrophobic free drugs (such as Ver-A) were exposed to a hydrophilic cell culture environment during treatment with harsh pH and temperature. Free drugs are more prone to degradation (such as hydrolysis) than the EV-encapsulated drugs, which could compromise the potency of free drugs during treatment. Overall, these data indicated that Ver-A and DM1 were involved in the regulation of proliferation and apoptosis of NET cells although the anticancer mechanisms need further investigation.

**Figure 3:**
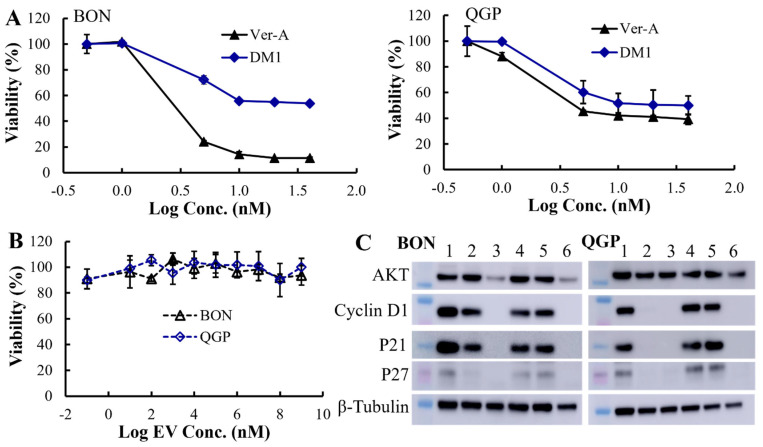
In vitro anticancer evaluation. (**A**) The anticancer cytotoxicity IC_50_ of free drug Ver-A (♦) and DM1 (▲) in BON and QGP cells (data represent mean ± SEM, *n* = 3). (**B**) No cytotoxicity of empty EV was detected. (**C**) Western blotting analysis of proliferation and apoptosis biomarkers in BON and QGP. 1: PBS control; 2: 5 nM Ver-A; 3: 10 nM Ver-A; 4: 10 nM DM1; 5: 20 nM DM1; and 6: 5 nM EV-Ver-A/DM1.

The authors state that the scientific conclusions are unaffected. This correction was approved by the Academic Editor. The original publication has also been updated.

## References

[1] Si Y., Guan J., Xu Y., Chen K., Kim S., Zhou L., Jaskula-Sztul R., Liu X.M. (2020). Dual-Targeted Extracellular Vesicles to Facilitate Combined Therapies for Neuroendocrine Cancer Treatment. Pharmaceutics.

